# Machine learning algorithms as early diagnostic tools for prolonged operative time in patients with fluorescent laparoscopic cholecystectomy: a retrospective cohort study

**DOI:** 10.3389/fsurg.2025.1582425

**Published:** 2025-06-23

**Authors:** Chu Wang, JunYe Wen, ZiYi Su, HanXiang Yu

**Affiliations:** ^1^ Graduate School, Hebei North University, Zhangjiakou, Hebei, China; ^2^ Department of Hepatobiliary Surgery, Hebei General Hospital, Shijiazhuang, Hebei, China; ^3^ School of Clinical Medicine, Hebei Medical University, Shijiazhuang, Hebei, China

**Keywords:** indocyanine green, cholecystectomy, laparoscopic, operative time, gallstones, predictive model

## Abstract

**Background:**

The purpose of this study was to explore the risk factors for prolonging the operative time of fluorescence laparoscopic cholecystectomy (LC). In addition, we aimed to construct predictive models to identify patients with potentially prolonged operative times (OT) using machine learning (Ml) methods.

**Methods:**

Clinical data of patients who underwent fluorescent LC for gallbladder stones in the Department of Hepatobiliary Surgery at our hospital from April 2023 to July 2024 were retrospectively analyzed, with the 75th percentile of operative time as the cut-off point. Parameters screened by univariate and multifactor analysis and LASSO regression were incorporated into the model, and the optimal model was analyzed and determined by integrating 11 Ml classification models.

**Results:**

The 85 min or more was defined as prolonged OT, and 29% (223/726) of patients had prolonged OT. The variables screened by univariate, multivariate analysis and lasso regression included type of cholecystitis, number of puncture ports, gallbladder adhesion, conservative antibiotic treatment before surgery, gallbladder thickness (mm). The above five parameters were incorporated into the Ml model. Comprehensive analysis revealed that the Light Gradient Boosting Machine (LightGBM) classification model was the optimal model, with the area under the curve (AUC) of the validation cohort was 0.876, the 95% confidence interval was 0.8139–0.938, the accuracy was 0.843, the sensitivity was 0.805, and the specificity was 0.857, with AUC of validation cohort was 0.876. The calibration curves showed good agreement between the actual and predicted probabilities of the LightGBM classification model; The decision curve analysis showed that the model had good net clinical benefit in most of the threshold probability range.

**Conclusions:**

We created a nomogram for assessing the risk of prolonged fluorescent LC time using the LightGBM classification model, which may help surgeon identify patients whose OT may be prolonged.

## 1 Introduction

About 6% of the global population suffers from gallbladder stones and the trend is increasing (1), with about 20% of them presenting with clinical symptoms such as epigastric pain, and requiring gallbladder removal for symptomatic gallbladder stones and asymptomatic gallbladder stones with risk factors for gallbladder cancer (2, 3). LC as the main operation for benign gallbladder diseases (4), it may be the most common surgical operations in the world (5). OT can influence LC outcomes, with up to 87 min of OT associated with postoperative superficial surgical site infections, organ-space infections, dehiscence, and septic shock, and prolonged hospitalization compared with 46 min (6). Traditional LC is mostly completed within 2 h (7). Ml can assist surgeons in making clinical decisions that are beneficial to patients (8), and has previously been used to assess the difficulty of LC but cannot infer the OT (9). In recent years, video imaging technology has made significant progress, and indocyanine green (ICG) fluorescence has been introduced into laparoscopic surgery (10). ICG can be rapidly discharged into the bile duct after intravenous injection, while near-infrared light penetrates 0.5–1 cm of human tissue and is absorbed by IGG and re-emitted at a specific wavelength, and the intraoperative fluorescence imaging system enables visualization of the extra-hepatic bile ducts (11, 12). Dissecting the Calot's triangle is a critical and time-consuming step in LC, and IGG fluorescence laparoscopy improves the visibility of the extrahepatic bile ducts, especially the Calot's triangle, compared to xenon white light imaging (12), allowing surgeons to easily identify critical anatomical landmarks in LC (13). Preoperative IGG injection reduces LC time to 21–46 min (14). In addition, inexperienced residents participation in LC will prolong the OT (15), while others believe that the ineffective guidance of the attending physician to the residents leads to the extension of the OT (16).

The aim of this study was to find out the factors that prolong the OT of fluorescent LC under the guidance of experienced chief physicians and deputy chief physicians. Developing an effective and practical predictive tool that would visualize the probability of the event, avoid or even eliminate the risk factors for prolonging the OT, reduce the occurrence of postoperative adverse events, and shorten the length of hospital stay. This study is not only applicable to traditional three or four port laparoscopy and single port laparoscopy, but also has important reference significance for 3D laparoscopy and robotic surgery.

## 2 Materials and methods

### 2.1 Participants

**Figure 1:**
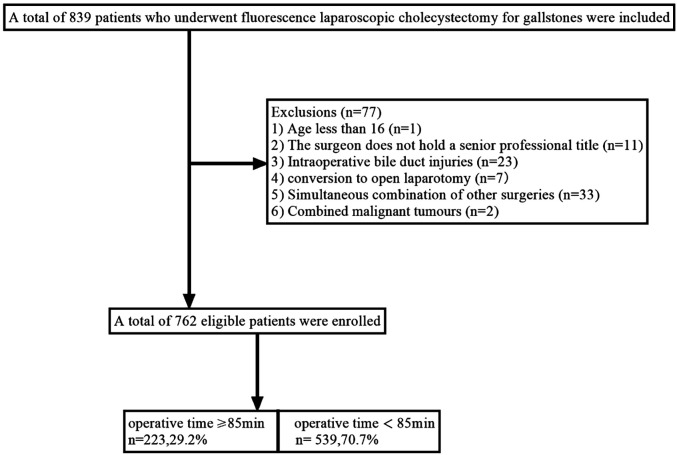
Flow chart of patient selection.

This study included patients who underwent fluorescent LC for gallstones in the Department of hepatobiliary surgery of Hebei General Hospital from April 1, 2023, to July 31, 2024. The chief physician or deputy chief physician served as the surgeon. The exclusion criteria are patients who meet one of the following characteristics. (1) Age less than 16 (*n* = 1); (2) The surgeon does not have a senior professional title (*n* = 11); (3) Intraoperative bile duct injuries (*n* = 23); (4) Conversion to open laparotomy (*n* = 7); (5) Simultaneous combination of other surgeries (*n* = 33); (6) Combined malignant tumours (*n* = 2). Finally, 762 patients were included in this study, and the patient selection process is shown in Figure 1.

### 2.2 Data collection

Baseline parameters of patients were collected, including age, gender, body mass index (BMI), and diabetes history. Preoperative information includes cholecystitis type, history of percutaneous transhepatic gallbladder drainage (PTGD), epigastric pain, conservative antibiotic treatment before surgery, white blood cell count, alanine aminotransferase, aspartate transaminase, bilirubin, gallbladder thickness and size. Intraoperative information included surgical methods (single-incision, three-port, and four-port laparoscopy) and gallbladder adhesion. The OT was calculated from skin incision to the end of skin suture. The 75th percentile of operative time (85 min) was used as the cut-off point, and an OT of 85 min or more was defined as prolonged OT.

### 2.3 Surgical procedures

In this study, three kinds of laparoscopic surgery methods (single-incision, three-port, and four-port laparoscopic) were used.

Single-incision laparoscopy: after successful anesthesia, the patient was placed in the supine position and disinfected three times according to the standard process. Make an arc incision at the lower edge of the umbilicus, insert a trocar with a diameter of 20 mm, place a camera to observe whether the abdominal cavity was damaged, and establish a 14 mmHg pneumoperitoneum with a CO2 pneumoperitoneum machine. The gallbladder and the Calot's triangle were isolated and exposed under the guidance of ICG fluorescence, followed by separation of the cystic duct using ultrasonic scalpel, closure of the cystic duct using bioabsorbable clips about 0.5 cm from the common bile duct, and the neck of the gallbladder using Hem-o-lock clips, with the cystic duct being cut between them. The cystic artery was clamped with bioabsorbable clip, and the distal end was cut off with ultrasonic scalpel. After removing the gallbladder through the abdominal incision, checked whether there was bleeding point and bile duct injury, wash the abdominal cavity with normal saline and anti-adhesion liquid, empty the CO2 in the abdominal cavity, and sutured the abdominal incision. 4-port laparoscope was performed using a 10 mm trocar in the umbilicus accompanied by three 5 mm trocars placed in the epigastric, middle right upper, or lower right lateral regions. Compared with four port-laparoscopy, three port laparoscopy omits the right lower abdominal lateral trocar.

### 2.4 Clinical features selected

The 17 clinical predictive variables were preprocessed, and the data with missing rate below 20% were processed by random forest interpolation, and the abnormal values in the data were cleared. Then, the predictive variables were standardized, and the clinical variables related to the research results were determined by univariate analysis. The odds ratio (OR) and 95% confidence interval (95% CI) of the variables were calculated, and then the normal group and the delay group were compared. Variables with significance in univariate analyses were included in multivariate analyses, and variables with significance in multivariate analyses were likely to be independent risk factors affecting the results. Lasso regression was used to filter variables to avoid poor fitting of the model, and variables with non-zero regression coefficients were included in the construction of the final prediction model.

### 2.5 Construction and performances assessment of the machine learning models

The patients were randomly divided into training cohort (534 cases) and validation cohort (228 cases) according to the ratio of 8:2. According to the selected prediction parameters, 11 Ml models such as Logistic Regression, Naive Bayes, Support Vector Machine, K-Nearest Neighbor, Random Forest, Extra Trees, eXtreme Gradient Boosting, LightGBM, Gradient Boosting, Adaptive Boosting, and Multilayer perceptron were constructed. 10-fold cross validation was performed on the training cohort to determine the hyperparameters in the final model. The models were evaluated and compared by sensitivity, specificity, positive predictive value, negative predictive value, accuracy, and area under the curve (AUC). The closer the AUC to 1, the better the performance of the model and best model was selected by plotting decision curve analysis (DCA). To improve the clinical practicability of the prediction model, we developed a nomogram. According to the contribution of each hyperparameter in the model to the results, each hyperparameter was scored, and then the total score was obtained by adding the scores. Through the total score, the probability of event occurrence can be calculated on the probabilit*y* axis. The nomogram visualizes the complex regression equation, making the prediction results more readable.

### 2.6 Statistical analysis

The categorical variables in the data were expressed in percentages and figures, the continuous variables in normal distribution were expressed in mean ± standard deviation (SD), and non-normally distributed data were expressed as median and Inter-Quartile Range (IQR). Categorical variables were compared by chi-square test or Fisher exact test, and continuous variables were compared by independent t test or Mann–Whitney *U*-test. Variables with *P* < 0.05 were further included in the analysis. SPSS (version 22.0).

## 3 Results

### 3.1 Clinical characteristics

Table 1 summarized the clinical characteristics of 762 patients. The average age of the patients was 58 years old. 55 cases (7.22%) had acute cholecystitis and 84 cases (11.02%) had acute exacerbation of chronic cholecystitis. There were 655 cases of gallbladder adhesion (85.96%). 40 cases (5.24%) underwent preoperative PTGD, and 239 cases (31.36%) received conservative antibiotic therapy before surgery. Of all the patients, single-incision laparoscopy was used in 27 cases (3.64%), four-port laparoscopy in 313 cases (41.07%), and three-port laparoscopy in 422 cases (55.38%). We randomly assigned these patients, 543cases (70%) of whom were assigned to the training cohort, and the remaining 228cases (30%) to the validation cohort.

**Table 1:** Clinical characteristics of patients.

Patient characteristics	Total (*n* = 762)	Normal (*n* = 539)	Delay (*n* = 223)	*p* value
Age (years), median (IQR)	58.00 (46.00, 67.00)	58.00 (46.00, 66.00)	58.00 (48.00, 68.00)	0.557
Gender, *n* (%)				0.563
Male	326 (42.78)	227 (42.12)	99 (44.39)	
Female	436 (57.22)	312 (57.88)	124 (55.61)	
BMI, Mean (SD)	26.72 (4.72)	26.44 (4.80)	27.39 (4.46)	**0** **.** **012**
Diabete, *n* (%)				0.077
No	652 (85.56)	469 (87.01)	183 (82.06)	
Yes	110 (14.44)	70 (12.99)	40 (17.94)	
Type of cholecystitis, *n* (%)				**<**.**001**
Chronic cholecystitis	623 (81.76)	471 (87.38)	152 (68.16)	
Acute cholecystitis	55 (7.22)	16 (2.97)	39 (17.49)	
Acute exacerbation of chronic cholecystitis	84 (11.02)	52 (9.65)	32 (14.35)	
Gallbladder Size (cm), median (IQR)	7.00 (6.50, 8.00)	7.00 (6.50, 8.00)	7.50 (6.30, 8.50)	**0**.**034**
Gallbladder thickness (mm), median (IQR)	3.00 (2.00, 4.00)	3.00 (2.00, 3.40)	4.60 (3.00, 6.00)	**<**.**001**
Gallbladder adhesion, *n* (%)				**<**.**001**
No	107 (14.04)	94 (17.44)	13 (5.83)	
Yes	655 (85.96)	445 (82.56)	210 (94.17)	
WBC (10^9^/L), median (IQR)	6.33 (5.13, 8.79)	6.08 (5.02, 7.89)	7.14 (5.49, 11.14)	**<**.**001**
Bilirubin (μmol/L), median (IQR)	15.00 (11.30, 21.70)	14.90 (11.60, 21.30)	15.20 (11.15, 22.55)	0.958
ALT (U/L), median (IQR)	21.30 (14.10, 41.10)	20.40 (14.00, 42.25)	22.50 (14.40, 38.50)	0.581
AST (U/L), median (IQR)	22.40 (17.70, 32.70)	22.50 (17.80, 32.40)	22.10 (17.40, 33.25)	0.344

Bold values represents statistically significant differences (*p*-value < 0.05).

### 3.2 Clinical characteristic differences between the study groups

**Figure 2:**
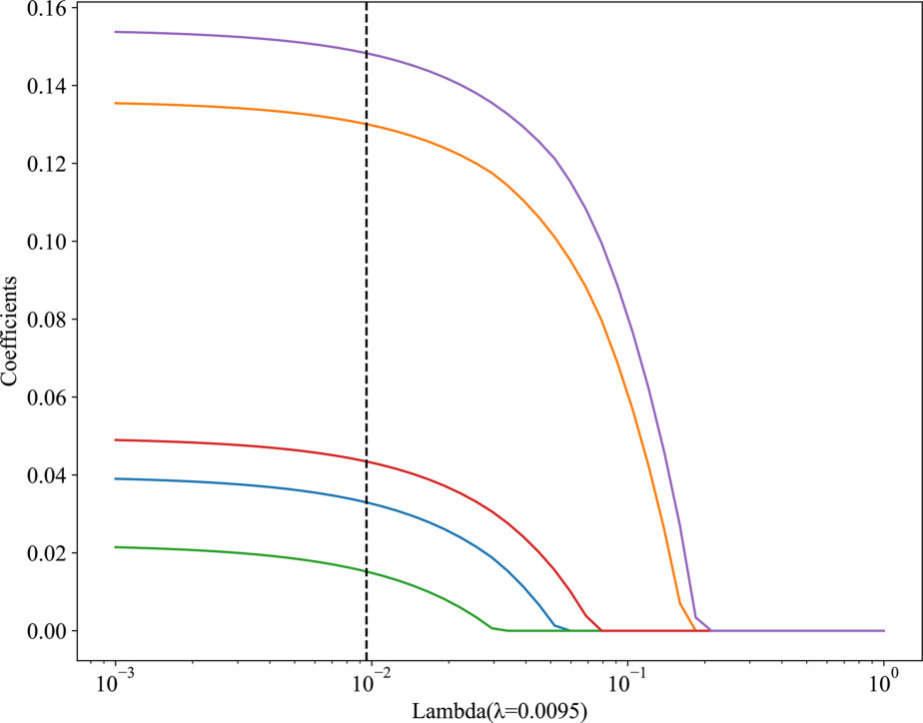
The results of the LASSO regression analysis. The abscissa *λ* represents regularization strengths, and the vertical axis represents the coefficients of features, as *λ* increases, features with non-zero coefficients at the dashed line have a greater impact on the results.

In univariate analysis (Table 2), the related factors included BMI, white blood cell count, gallbladder adhesion, gallbladder size, gallbladder thickness, acute cholecystitis, acute exacerbation of chronic cholecystitis, conservative antibiotic treatment before surgery, PTGD, single-incision and four-port surgical methods. In multivariate analysis, gallbladder adhesion, acute cholecystitis, conservative antimicrobial treatment before surgery, single-incision and four-port were independent predictors of prolonged OT (Table 2). Lasso regression was used to analyze the features with significant differences in the training cohort, which can also compress the variable coefficients, prevent over fitting, and solve the problem of collinearity. Finally, five features with non-zero regression coefficients were selected for model construction, including type of cholecystitis, number of puncture ports, gallbladder adhesion, conservative antibiotic treatment before surgery, gallbladder thickness (mm) (Figure 2).

**Table 2:** Univariate and multivariate logistic regression results.

Variables	Univariate analysis		Multivariate analysis	
	OR (95% CI)	*P* value	OR (95% CI)	*p* value
Age (years)	1.00 (0.99–1.01)	0.588		
Gender (male)	0.91 (0.67–1.25)	0.563		
BMI (Kg/m^2^)	1.04 (1.01–1.08)	**0**.**018**	1.04 (0.99–1.09)	0.120
WBC (10^9^/L)	1.11 (1.06–1.16)	**<**.**001**	0.99 (0.95–1.04)	0.733
Diabetes	1.46 (0.96–2.24)	0.078		
Epigastric pain	1.34 (0.90–2.01)	0.149		
Gallbladder adhesion	3.41 (1.87–6.23)	**<**.**001**	2.04 (1.03–4.07)	**0**.**042**
Gallbladder Size (cm)	1.13 (1.02–1.25)	**0**.**021**	0.91 (0.78–1.06)	0.216
Gallbladder Thickness (mm)	1.89 (1.67–2.14)	**<**.**001**	1.76 (1.54–2.01)	**<**.**001**
Type of cholecystitis				
Chronic cholecystitis	1.00 (Reference)		1.00 (Reference)	
Acute cholecystitis	7.55 (4.10–13.90)	**<**.**001**	2.80 (1.34–5.83)	**0**.**006**
Acute exacerbation of Chronic cholecystitis	1.91 (1.18–3.07)	**0**.**008**	1.50 (0.86–2.62)	0.151
Conservative antibiotic treatment before surgery	3.27 (2.35–4.55)	**<**.**001**	1.97 (1.32–2.94)	**<**.**001**
PTGD	2.69 (1.43–5.07)	**0**.**002**	1.03 (0.40–2.67)	0.948
Number of puncture ports				
Three-port	1.00 (Reference)		1.00 (Reference)	
Four-port	4.33 (3.06–6.12)	**<**.**001**	2.74 (1.82–4.11)	**<**.**001**
Single-incision	15.69 (6.38–38.61)	**<**.**001**	21.45 (8.24–55.86)	**<**.**001**

Bold values represents statistically significant differences (*p*-value < 0.05).

### 3.3 Construction of the predictive model

**Figure 3:**
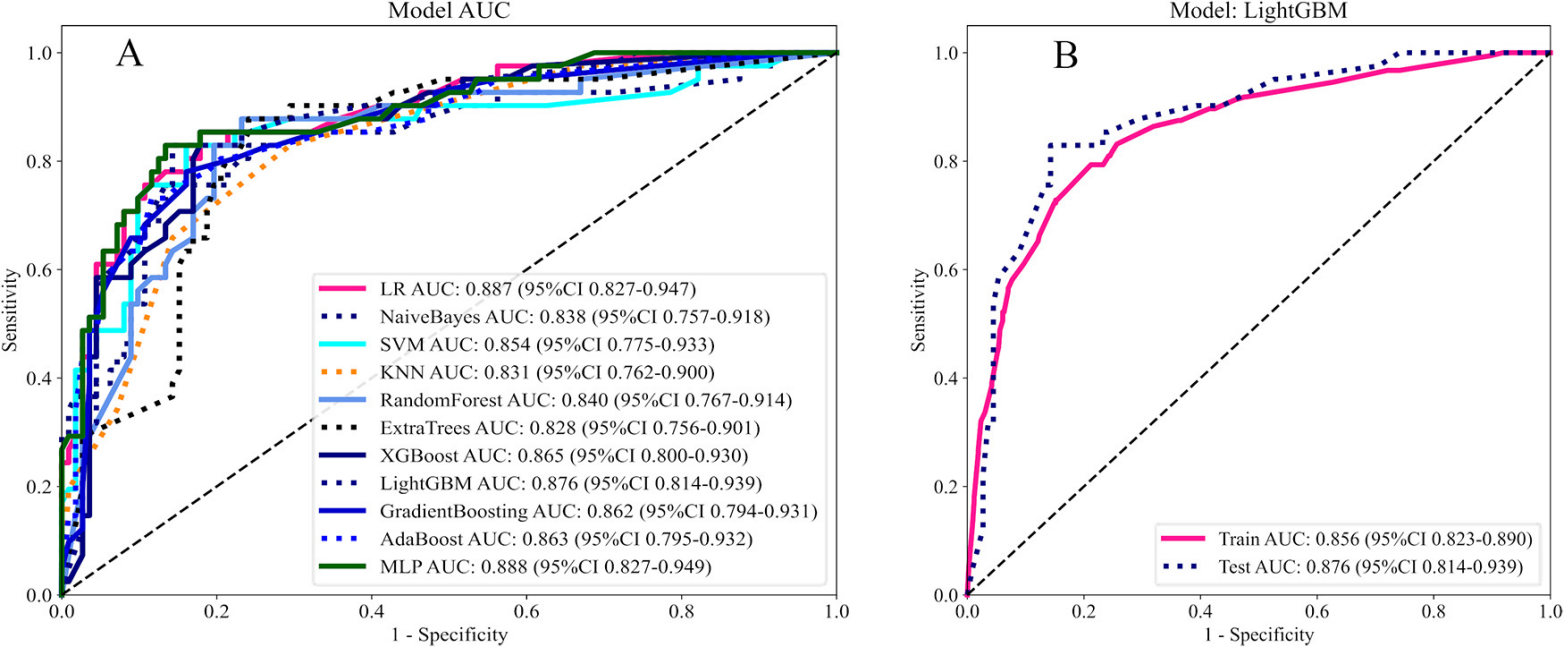
**(A)** AUC of 11 Ml models. **(B)** AUC of LightGBM model. Model performance is evaluated by training cohort, validation cohort.

**Figure 4:**
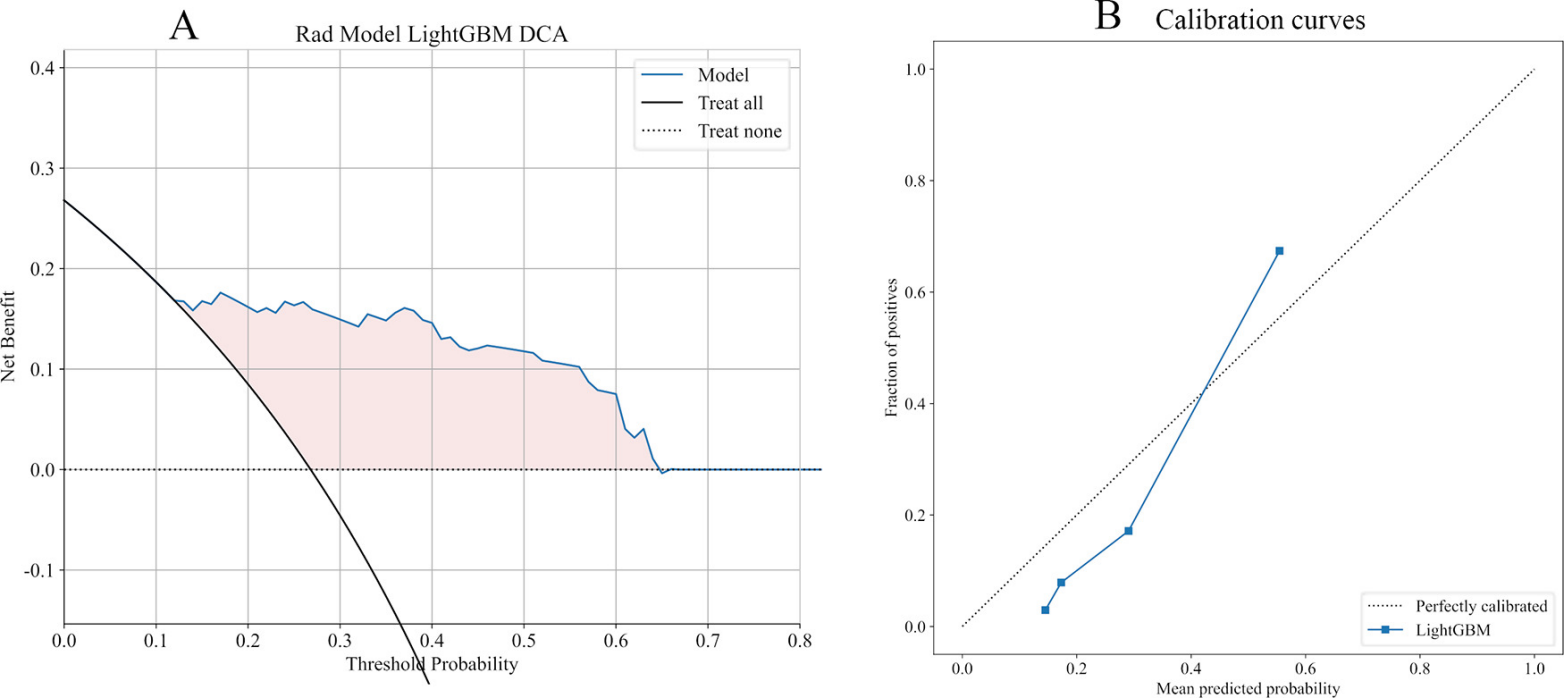
**(A)** DCA for LightGBM model. The curve shows that the model has a good clinical net benefit in most of the threshold probability range. **(B)** The dotted line represents the actual probability, and the blue line represents the predicted probability of the model, with closer proximity between the two representing better predictive performance.

11 Ml models were constructed using predictors, and the predictive performance of each Ml model was compared in the training and validation cohort (Table 3), with satisfactory results for each model in the validation cohort (Figure 3A). Combined with the analysis of model performance (Figure 3B) and DCA (Figure 4A), LightGBM model is an ideal model for identifying patients with potential prolonged OT, with an AUC value of 0.876 (95% CI:0.813–0.938), and its SEN (80%), SPE (85%), ACC (84%), positive predictive value (67%), negative predictive value (92%). The calibration curves for the LightGBM prediction model are as follows (Figure 4B), and the curves show that the actual probabilities in the validation cohort are in good agreement with the predicted probabilities.

**Table 3:** Model performance.

Model	Training cohort				Validation cohort			
	AUC (95% CI)	ACC (%)	SEN (%)	SPE (%)	AUC (95% CI)	ACC (%	SEN (%)	SPE (%)
LR	0.840 (0.806–0.874)	0.742	0.859	0.692	0.887 (0.827–0.947)	0.817	0.805	0.821
Naive Bayes	0.812 (0.773–0.849)	0.749	0.772	0.739	0.838 (0.757–0.917)	0.830	0.732	0.866
SVM	0.844 (0.809–0.879)	0.808	0.755	0.830	0.854 (0.774–0.932)	0.830	0.805	0.839
KNN	0.853 (0.821–0.884)	0.811	0.609	0.897	0.831 (0.761–0.900)	0.804	0.659	0.857
Random Forest	0.907 (0.881–0.933)	0.845	0.788	0.869	0.840 (0.766–0.913)	0.791	0.854	0.768
Extra Trees	0.919 (0.895–0.942)	0.855	0.793	0.881	0.828 (0.755–0.900)	0.784	0.854	0.759
XGBoost	0.878 (0.847–0.908)	0.816	0.745	0.846	0.865 (0.799–0.930)	0.824	0.829	0.821
LightGBM	0.856 (0.823–0.889)	0.811	0.728	0.846	0.876 (0.813–0.938)	0.843	0.805	0.857
Gradient Boosting	0.857 (0.823–0.890)	0.808	0.745	0.834	0.862 (0.794–0.930)	0.817	0.756	0.839
AdaBoost	0.846 (0.812–0.880)	0.777	0.793	0.769	0.863 (0.794–0.932)	0.830	0.634	0.902
MLP	0.851 (0.817–0,884)	0.790	0.766	0.800	0.888 (0.827–0.949)	0.850	0.805	0.866

LR, Logistic Regression; SVM, Support Vector Machine; KNM, K-Nearest Neighbor; Extra Trees, Extremely randomized trees; XGBoost, eXtreme Gradient Boosting; AdaBoost, Adaptive Boosting; MLP, Multilayer Perceptron.

### 3.4 Clinical value of the nomogram

**Figure 5:**
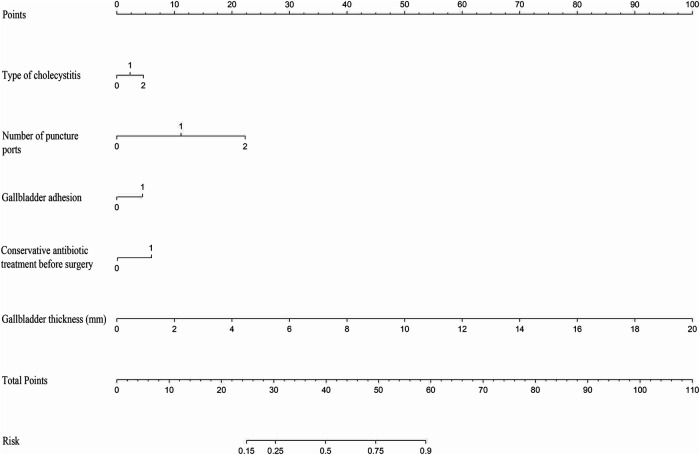
Use nomogram to determine the possibility of prolonged operative time. In the type of cholecystitis, 1 represents acute cholecystitis and 2 represents acute exacerbation of chronic cholecystitis; in number of puncture ports, 1 represents four holes and 2 represents single-incision.

We established a nomogram (Figure 5) to predict prolonged OT, assigning points to the predictors in the nomogram, with gallbladder thickness scoring the highest (0–100 points), followed by number of holes (0–22 points), conservative antibiotic treatment before surgery (6 points), type of cholecystitis (0–5 points), and gallbladder adhesions (4 points). The probability of prolongation of the operative time for each patient was visually estimated by summing the points for the corresponding variables and locating the corresponding score on the total score axis.

## 4 Discussion

ICG fluorescence imaging in cholecystectomy has the characteristics of fast, easy and safe exposure of the bile ducts, and the learning curve is similar to the traditional LC, which is expected to become the mainstream surgical procedure for cholecystectomy (17–19). There have been previous prediction models for cholecystectomy OT. Cornelius et al. used a classification tree model to predict LC OT, incorporating four factors: gender, BMI, American Society of Anesthesiologists Classification, and abnormal liver function, but the model fit was poor (20). Bharamgoudar et al. developed a scoring tool to predict operative time for elective LC based on ten risk factors such as gallbladder thickness, common bile duct diameter, etc., assigning a score to each factor, with higher total scores being associated with longer OT (21). Bourgouin et al. developed a simple and reliable scoring tool based on preoperative variables: gender, previous cholecystitis attack, neutrophil count, fibrinogen, and alkaline phosphatase, which had the best predictive effect with an AUC of 0.8 (22). Previous studies have considered the effect of the absence of ICG fluorescence imaging and operator experience on OT.

Similar to previous studies, we found that increased BMI was a risk factor for prolonging the OT (23, 24). Obesity and abdominal fat cause the umbilicus downward displacement and difficult in identifying the umbilical fascia, leading to obvious difficulty in the placement of the umbilical port and restricted port movement (25, 26). In addition, fat accumulation may result in the inability of near-infrared light to penetrate the bile ducts and the inability of IGG to expose the cystic ducts and extrahepatic bile ducts (11, 27). Fernando found that each 1-unit increase in BMI reduced visualization of the accessory bile duct by 10% and the cystic ducts by 3% prior to dissection, and that even after dissection some biliary structures remained less well visualized, with an overall average reduction in biliary visualization of 6% per unit of BMI increase (12). Lean patients had no effect on LC time (28), and obese patients receiving a very low-calorie diet (947 kcal per day) for a fortnight prior to surgery made the Calot's triangle more recognisable and reduced OT by 20% (29), which confirmed the impact of BMI on surgery from the side.

About 12% of gallbladder stones progress to acute cholecystitis (30), and according to the Tokyo Guidelines 2018, elevated white blood cells indicate the presence of acute cholecystitis (31). The recognized optimal time for surgery in acute cholecystitis is within 3 days (32–36), however, patients often miss the optimal time for surgery when they attend the clinic (the average waiting time for operation in this study is 4.03 days) (37). In the early stages of acute inflammation, adhesions in the plane of oedema around the gallbladder are loose, and over time, inflammation and oedema are replaced by severe fibrotic adhesions between the gallbladder and the surrounding tissues, and separation of the gallbladder becomes extremely difficult (38). Solid adhesion between gallbladder and surrounding organs and greater omentum can be seen in the course of more than 7 days (39), and the adhesion of gallbladder delayed for 3 weeks is more dense (33). In addition, ICG fluorescence is difficult to observe in acute cholecystitis because of edema and severe inflammation of the tissues surrounding the gallbladder, the cystic duct, and the hepatic pedicle (13). Fernando et al. found that bile duct visualization was 50% to 100% higher in mild inflammation than in moderate to severe inflammation (12). Lack of intraoperative IGG fluorescence to guide the surgery resulted in prolonged time.

PTGD is a transitional treatment for acute cholecystitis with delayed cholecystectomy. Compared with emergency cholecystectomy, patients with acute cholecystitis of more than 3 days' course who undergo PTGD followed by cholecystectomy have a shorter operative time (40). However, prolonged drainage produces more fibrous exudates and adhesions, and preoperative PTGD of more than 8 weeks can cause a significant increase in the difficulty and duration of surgery (41). Presently, there is no consensus on the optimal timing for LC after PTGD. Some studies suggest that the OT of cholecystectomy within 3–5 days (42), 7 days (43), 7–26 days (44), and 35 days (45) after PTGD is shorter, and duration of PTGD more than 60 days will increase the risk of cholecystitis recurrence and tube detachment (45). It is suggested that PTGD should not exceed 60 days. Without extubation before operation, the OT will be also prolonged (46). Wei et al. found that Elevated preoperative inflammatory markers increased the risk of LC for more than 90 min after PTGD (47). In a randomized controlled trial, compared with PTGD, ENBD (Endoscopic Naso-gallbladder Drainage) has lighter gallbladder inflammation, less intraoperative adhesion and shorter OT, ENBD can be used as a safe and effective treatment to replace PTGD (48).

Normal gallbladder thickness is less than 3 mm on ultrasonography, and varying degrees of gallbladder thickening can be seen in chronic cholecystitis and acute cholecystitis (49, 50). Nikolaos et al. analysed 1,089 cholecystectomy patients and found that thickening of the gallbladder caused abnormal distortion of the Calot's triangle and difficulty in grasping the gallbladder, making separation of the gallbladder from the gallbladder fossa difficult, and that for every 1-mm increase in the thickness of the gallbladder, the operative time increased by 4.2 min (51). Abdulrahman believe that the thickening of gallbladder wall may be related to the increase of intraoperative blood loss (52), which requires more time to stop bleeding.

Acute cholecystitis is often converted from chronic cholecystitis (53) and always relapse frequently after remission (54). Approximately 13% of patients relapsed around 100 days after conservative antibiotic treatment (55), and another study found that patients with acute cholecystitis who underwent delayed LC had a relapse rate of up to 44.6%, with a median time to relapse of 2.8 months (56). Recurrent episodes of cholecystitis can cause thickening of the gallbladder and dense adhesions at the Calot's triangle and gallbladder fossa, resulting in increased difficulty in LC (30, 57). Acute exacerbations of chronic cholecystitis and a history of conservative treatment with antibiotics are indicative of cholecystitis attacks. Patients undergoing conservative treatment often choose to refuse surgery for other reasons such as medical background and fear of surgery (54), which may result in patients deciding to undergo surgery when they have already experienced a higher number of recurrences. The nomogram we created similarly suggest that patients receiving conservative antibiotic therapy have longer operative times than those with acute exacerbation of chronic cholecystitis. The more recurrent cholecystitis, the longer the OT.

Cholecystitis with gallbladder adhesion to omentum or bowel can make laparoscopic surgery difficult (58). The presence of adhesions restricts the surgical field of view and obscures critical anatomy, and attempts to separate these adhesions take more time so as not to injure the surrounding vital organs and tissues (59). Kapoor et al. used acoustic radiation force impulse to acquire images of the gallbladder fundus, body, and neck region, and the virtual touch imaging function to indicate the adhesion site in red, which can accurately detect gallbladder adhesions (60).

Gallbladder distension is also one of the causes of difficulty in LC (61). A distended gallbladder is not easily grasped because it tends to slip away, and the presence of pericholecystic inflammation makes the gallbladder wall friable and oedematous, thus making grasp difficult. A distended gallbladder can make it difficult to remove the specimen through a small incision, so the gallbladder needs to be aspirated and the epigastric incision lengthened (26).

Like the study by Lin et al, three-port laparoscopic cholecystectomy took the shortest time, followed by four-port LC, and single-port LC was the longest (62). The shorter time for three-port LC compared to four-port is associated with less time spent on port establishment and subsequent closure (63). Several studies have confirmed that higher surgical difficulty is the main reason for significantly longer single-port LC times (64–66). Improved surgical and imaging modalities may reduce OT. Yun et al. concluded that 3D laparoscopic imaging may reduce OT by providing a better view and easier identification of the calot's triangle and gallbladder structures than 2D (67). Fundus first laparoscopic cholecystectomy is similar to open surgery and reduces the OT in patients with difficult cholecystectomies such as acute cholecystitis and tight adhesions around the Calot's triangle (68, 69).

Nomograms can guide the clinician's surgical decisions. Patients with acute cholecystitis should be operated on as early as possible, and those who miss the optimal time for surgery should be operated on within 60 days of PTGD. For obese patients undergoing elective surgery, low calorie diet should be given for two weeks before cholecystectomy. Preoperative ultrasonography to clarify gallbladder adhesions is necessary to assess the difficulty of surgery. As cholecystitis recurs frequently, patients should be informed in detail that cholecystitis still has a high risk of recurrence after conservative treatment and that the gallbladder should be removed early.

Unlike previously, this study did not find gender, and age to be associated with prolonged OT. This may be due to update of surgical equipment and improved surgeon expertise and surgical techniques. In addition, our study has some limitations; as a retrospective study, we may have missed some important clinical factors associated with prolongation surgery and secondly, there is a possibility of bias. Therefore, predictive models need to be externally validated in larger retrospective studies to confirm model prediction accuracy.

## 5 Conclusion

In summary, this study identified factors associated with prolonged fluorescent laparoscopic cholecystectomy time, and for the first time established a nomogram, which can objectively and individually predict the duration of surgery. This study may help clinicians to better improve preoperative preparation, rationally arrange surgery, and reduce patients' postoperative complications and hospitalization time.

## Data Availability

The raw data supporting the conclusions of this article will be made available by the authors, without undue reservation.
